# Presymptomatic treatment of classic late-infantile neuronal ceroid lipofuscinosis with cerliponase alfa

**DOI:** 10.1186/s13023-021-01858-6

**Published:** 2021-05-14

**Authors:** J. Schaefers, L. J. van der Giessen, C. Klees, E. H. Jacobs, S. Sieverdink, M. H. G. Dremmen, J. K. H. Spoor, A. T. van der Ploeg, J. M. P. van den Hout, H. H. Huidekoper

**Affiliations:** 1 Department of Pediatrics, Center for Lysosomal and Metabolic Diseases, Erasmus MC, University Medical Center Rotterdam, PO Box 2040, 3000 CA Rotterdam, The Netherlands; 2 Department of Pediatric Physiotherapy, Erasmus MC, University Medical Center Rotterdam, Rotterdam, The Netherlands; 3 Department of Child and Adolescent Psychiatry/Psychology, Erasmus MC, University Medical Center Rotterdam, Rotterdam, The Netherlands; 4 Department of Clinical Genetics, Erasmus MC, University Medical Center Rotterdam, Rotterdam, The Netherlands; 5 Department of Radiology and Nuclear Medicine, Erasmus MC, University Medical Center Rotterdam, Rotterdam, The Netherlands; 6 Department of Pediatric Neurosurgery, Erasmus MC, University Medical Center Rotterdam, Rotterdam, The Netherlands

**Keywords:** Late-infantile neuronal ceroid lipofuscinosis, CLN2 disease, Tripeptidyl peptidase, Presymptomatic, Cerliponase alfa, Enzyme replacement therapy, Intracerebroventricular, Lysosomal storage disorder

## Abstract

**Background:**

Neuronal ceroid lipofuscinosis type 2 (CLN2 disease) is a rare rapidly progressive neurodegenerative disorder, resulting in early death. Intracerebroventricular enzyme replacement therapy (ERT) with cerliponase alfa is now available and has shown to delay disease progression in symptomatic patients. It is yet unknown if cerliponase alfa can prevent disease onset in presymptomatic patients.

**Results:**

We evaluated the effect of 2 years of intracerebroventricular ERT in two siblings with CLN2 disease, one symptomatic (age 47 months) and one presymptomatic (age 23 months) at treatment start, using the CLN2 Clinical Rating Scale (CLN2 CRS), Gross Motor Function Measure-66 (GMFM-66) for motor function, Bayley Scales of Infant and Toddler Development, 3rd Edition, Dutch (BSID-III-NL) for neurocognitive development, brain MRI, and visual evoked potentials (VEP), electroretinogram (ERG) and retinoscopy for visual function. On the CLN2 CRS patient 1 showed a decline from 3 to 2 in the combined motor and language score due to regression in language use (CLN2 CRS total score after 2 years of treatment: 8), whereas a decline of 2 or more points in the combined motor and language score would be expected without treatment. Patient 2 retained the maximum score of 3 in all 4 subdomains (CLN2 CRS total score after 2 years of treatment: 12). The GMFM-66 total score declined from 46 to 39 in patient 1 and showed an age-appropriate increase from 66 to 84 in patient 2. Cognitive-developmental age decreased from 24 to 11 months in patient 1, whereas an increase in cognitive-developmental age from 21 to 39 months was seen in patient 2. Cerebral and cerebellar atrophy observed on MRI in patient 1 at age 42 months (before treatment) was not observed in patient 2 at age 48 months (after 2 years of treatment).

**Conclusion:**

We show that cerliponase alfa is able to delay the onset of symptoms when treatment is started in a presymptomatic stage of CLN2 disease. Our results advocate the start of treatment at an early age before symptom onset, but should be confirmed in a larger cohort study.

## Background

Neuronal ceroid lipofuscinosis type 2 (CLN2 disease; OMIM#204500) is an autosomal recessive disease, caused by deficiency of the lysosomal enzyme tripeptidyl peptidase (TPP1; EC 3.4.14.9) [1, 2]. The natural substrate and function of TPP1 are unknown, but its deficiency leads to the accumulation of auto fluorescent storage material (ceroid lipofuscin) in the lysosomes and neuronal loss [3]. In the classic late-infantile form of CLN2 disease this results in a rapidly progressive neurodegenerative disorder with a highly homogeneous course of disease. Typically, patients present between the age of 2 and 4 years with an unprovoked seizure, often a language delay will already be present. Thereafter, patients exhibit a rapid decline in cognitive, language, motor and visual function, with complete loss of motor and speech functions by 6–7 years of age, followed by blindness and premature death between 8 and 13 years [4–7]. More than half of the patients are either homozygous or compound heterozygous for the splicing mutation c.509-1G > A and/or the nonsense mutation c.622C > T [4, 5, 8, 9]. Progression and onset of disease is largely homogeneous and predictable for several genotypes [10–12].

Until 2017, only supportive treatment with a multi-disciplinary approach was available for patients with CLN2 disease [13, 14]. However in 2017, the first intracerebroventricular enzyme replacement therapy with cerliponase alfa, a recombinant human TPP1 enzyme, was approved based on the results of the pivotal trial conducted in 23 CLN2 patients [15, 16]. It was shown that patients treated with cerliponase alfa had a slower decline in the adjusted motor-language score of the CLN2 clinical rating scale, the primary outcome measure of the trial, compared to historical controls (0.38 ± 0,10 vs 2.06 ± 0.15 points) after 48 weeks of treatment [15]. Although these results are very promising, long term efficacy has yet to be demonstrated. In addition, the potential of delaying disease onset when treatment is started in a presymptomatic stage of the disease remains to be shown and is currently being investigated in a phase 2 trial (NCT02678689).

In this paper, we report on the clinical outcome in two siblings with classic late-infantile CLN2 disease treated with cerliponase alfa for 2 years. The youngest sibling was started on treatment before the onset of clinical symptoms.

## Results

### Case descriptions before initiation of treatment

#### Patient 1

The index patient was the eldest daughter of non-consanguineous Dutch parents. She was born at term after an uncomplicated pregnancy and delivery. Her motor development was delayed, related to hip dysplasia which was treated with a Pavlik harness after which she became a bottom-shuffler and started walking at age 26 months. At age 30 months she was able to run and make knob puzzles. She spoke two-word sentences at age 24 months, and was able to speak three-word sentences and name objects at age 30 months. From age 39 months onward her parents started to notice the development of a tremor in her right arm and leg, and a loss in vocabulary. In the months thereafter she developed hypertonia of her legs, eventually leading to loss of the ability to stand and walk without support at the age of 43 months, the inability to produce two-word sentences or name objects at age 48 months, and eating difficulties.

Her first tonic–clonic seizure occurred at age 42 months, which was treated with levatiracetam. In retrospect her parents observed frequent absence seizures prior to this. They also noticed a change in behavior. She had always been a happy child, but at age 40 months she became more irritable, cried often and seemed frustrated. She also developed a sleeping disorder where she woke up the middle of the night and remained awake thereafter.

Whole exome sequencing was performed at the age of 44 months. This revealed the presence of the two most prevalent pathogenic variants in *TPP1*, c.622C > T; (p.Arg208*) and c.509-1G > C. Tripeptidyl peptidase deficiency was confirmed in leucocytes (TPP1 activity was 20.3 nmol/h/mg; control range 125–340 nmol/h/mg). At age 47 months treatment with cerliponase alfa was started at the Center for Lysosomal and Metabolic Diseases, Erasmus MC University Medical Center.

#### Patient 2

This patient is the younger sibling of patient 1. He was born at term after an uncomplicated pregnancy and delivery. He started walking at age 14 months and was able to run and climb the stairs soon thereafter. His fine motor skills were age appropriate. He started using single words before his first birthday and he spoke two-word sentences, with a normal vocabulary and good non-verbal communication skills at age 21 months. He was diagnosed with CLN2 disease based on the diagnosis of his sister: he had the same *TPP1* genotype and tripeptidyl peptidase deficiency in leucocytes was also confirmed (TPP1 activity: 16.9 nmol/h/mg; control range 125–340 nmol/h/mg). He did not exhibit any signs of epilepsy. He was started on treatment with cerliponase alfa at age 23 months. At that time there were no symptoms of CLN2 disease.

### Assessment of treatment efficacy

#### CLN2 clinical rating scale (CLN2 CRS)

**Fig. 1:**
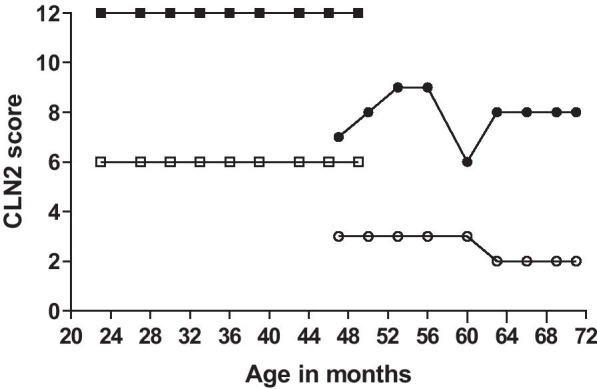
CLN2 clinical rating scale (CLN2 CRS) in patient 1 (bullets) and patient 2 (boxes) during two years of treatment with cerliponase alfa. Closed bullets/boxes represent total sum score of all four domains and open bullets/boxes represent the sum score of the motor and language domain

At baseline (age 47 months) patient 1 had a score of 3 on the combined motor and language scale and a CLN2 CRS total score of 7. After 15 months of treatment she lost 1 point on the language domain (CLN2 CRS combined motor-language score: 2). The score for her epilepsy fluctuated over time between 0 and 3, resulting in a CLN2 CRS total score fluctuating between 6 and 9 (Fig. 1).

Patient 2 had maximum scores on all subdomains of the CLN2 CRS at baseline (CLN2 CRS total score: 12), which remained the same over the course of treatment (Fig. 1).

#### Evaluation of gross motor function

**Fig. 2:**
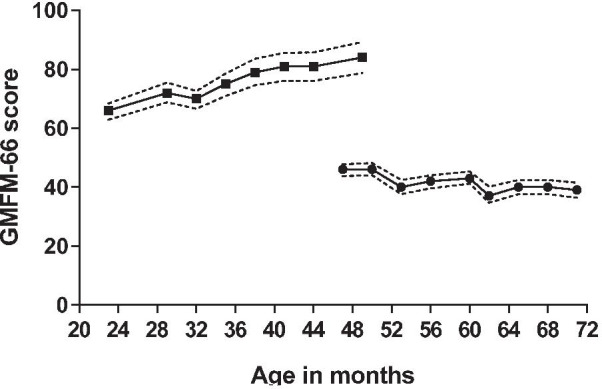
Gross motor function measure-66 (GMFM-66) total scores in patient 1 (bullets) and patient 2 (boxes) during two years of treatment with cerliponase alfa. Dotted lines represent 95% confidence intervals

Patient 1 lost her ability to walk without support at the age of 43 months. At baseline she had severe hypertonia, most prominent in her lower limbs, for which a combination of trihexyphenidyl and baclofen was started. She also received night splints to correct contractures of her feet. This resulted in some improvement of her mobility, where she was able to crawl again. Her Gross Motor Function Measure-66 (GMFM-66) total score at baseline was 46 (95% CI 43.7–47.8) and decreased to 39 (95% CI 36.4–41.5) after 2 years of treatment (Fig. 2).

Patient 2 had age-appropriate gross motor skills at baseline (GMFM-66 total score 66 (95% CI 62.9–68.4) and he continued to have a normal gross motor development thereafter. At the age of 49 months he was still gaining motor skills, while patient 1 at the same age had already lost many of her motor abilities (Fig. 2). His GMFM-66 total score after 2 years of treatment was 84 (95% CI 78.8–89.3).

#### Neuropsychological development

At baseline (age 46 months) patient 1 had, based on the the Bayley Scales of Infant and Toddler Development, 3rd Edition, Dutch version (BSID-III-NL), a cognitive-developmental age equivalent to 24 months and receptive and expressive communication skills equivalent to a developmental age of 27 months. After 2 years of therapy (age 72 months) her cognitive-developmental age was equivalent to 11 months and her receptive and expressive communication skills were equivalent to a developmental age of 10 and 17 months, respectively.

At baseline (age 22 months) patient 2 had age-appropriate scores on both the cognitive and language scales of the BSID-III-NL. At age 29 months his cognitive and communication skills were equivalent to a developmental age of 27 months. At age 48 months, after 2 years of treatment, his cognitive-developmental age was assessed to be 39 months, but we were not able to reliably assess his language skills due to lack of sufficient cooperation during testing. We therefore estimated his language skills according to the BSID-III-NL based on parent reports. His receptive and expressive communication skills seemed equivalent to a developmental age of 39–42 months. He had just started attending a regular primary school.

#### Visual impairment

Parents did not notice any visual impairments of both patients during the study. Both patients had a normal ophthalmological examination at baseline and after 2 years of treatment. In patient 1 visual evoked potentials (VEP) performed at baseline and at regular intervals during 18 months of treatment showed giant potentials consistent with CLN2 disease [17]. Due to agitation it was technically impossible to repeat a VEP at 24 months of treatment. Electroretinography (ERG) at 6 months of treatment showed normal latency times, an ERG could not be done at baseline as well as during 6–24 months of treatment due to agitation.

In patient 2 a VEP/ERG was not performed at baseline. Subsequent VEPs showed bilateral prolonged p100 latency with normal amplitude. No giant potentials were seen during 2 years of treatment. ERGs were normal.

#### Neuroimaging

**Fig. 3:**
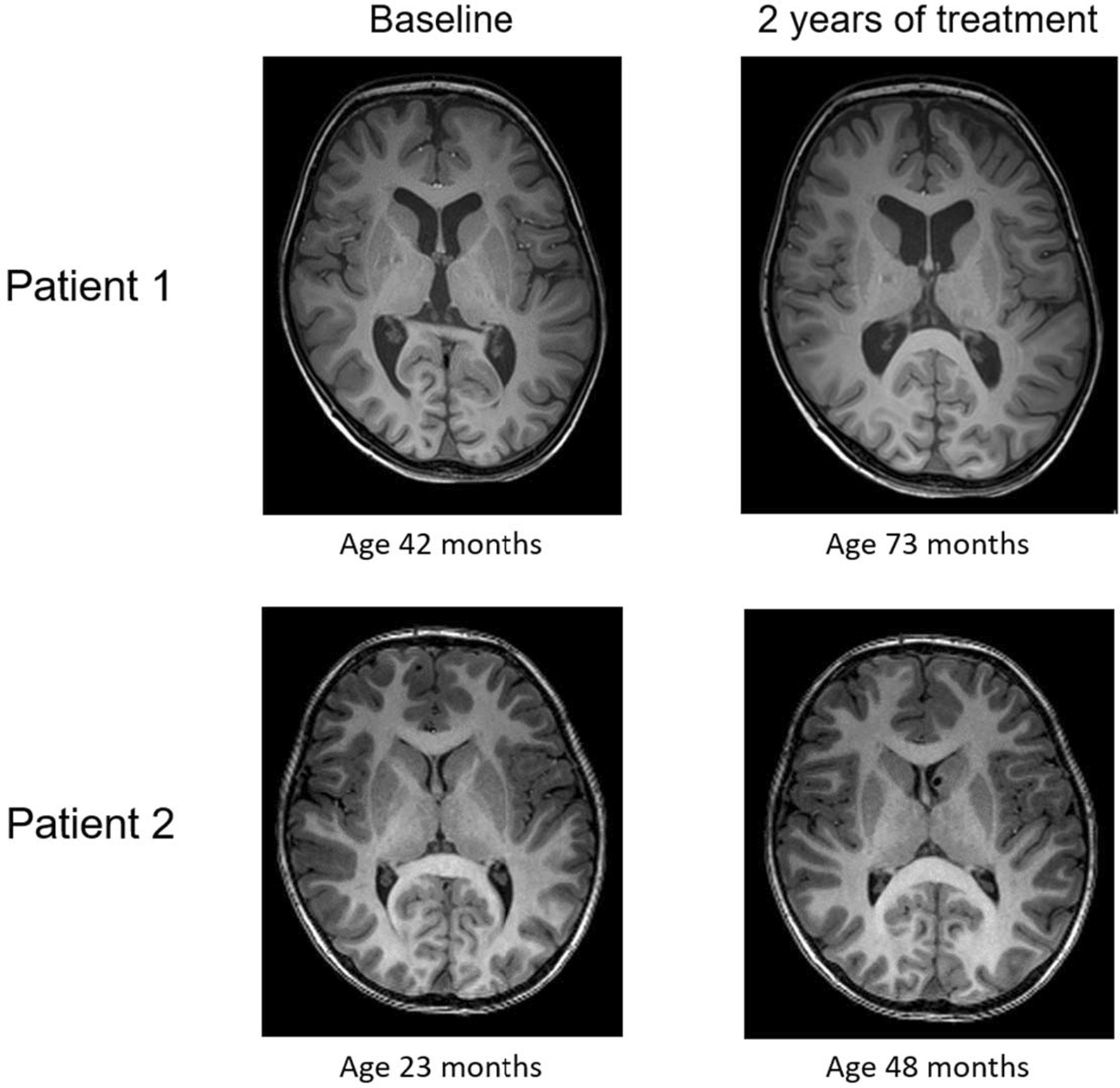
Brain MRI at baseline and after approximately two years of treatment with cerliponase alfa. Axial T1-weighted MR images at the level of the lateral ventricles. In patient 1 cerebral atrophy with ex vacuo ventricular dilatation was already present at 42 months of age (baseline), whereas no cerebral atrophy was present in patient 2 at 48 months of age after 2 years of treatment with cerliponase alfa

The baseline brain magnetic resonance imaging (MRI) of patient 1 showed mild cerebral and cerebellar atrophy with concomitant ex vacuo dilatation of the ventricle system (Fig. 3), and T2 hyperintense signal abnormalities of the periventricular white matter, consistent with CLN2 disease [6, 17, 18]. The brain MRI after 2 years of treatment showed progressive cerebral and cerebellar atrophy with progressive ex vacuo dilatation of the ventricles (Fig. 3) and mild progression of the periventricular white matter abnormalities.

Patient 2 had a normal brain MRI at baseline. The brain MRI after 2 years of treatment (at age 48 months) showed no signs of cerebral or cerebellar atrophy (Fig. 3), but did show a mild hyper intense T2 signal of the periventricular white matter at the occipital horns, which was considered to be related to terminal myelination zones.

#### Epilepsy

At baseline patient 1 exhibited a mixed type of epilepsy with tonic–clonic seizures, myoclonic seizures, atonic seizures and absences. Her anticonvulsants were adapted over time. With the combination of lamotrigine, levatiracetam and clobazam, she had multiple short (duration up to 20 s) partial complex seizures (both tonic and atonic) and/or absence seizures per day with only sporadic myoclonic seizures at her last evaluation. The last tonic–clonic grand mal seizure occurred at age 59 months. No aggravation of the seizures was noticed during infusion of cerliponase alfa nor in the days thereafter.

Patient 2 did not have any signs of epilepsy during 2 years of treatment and remains free of epilepsy to date.

#### Behavior and sleeping disorders

Before the start of therapy, the behavior of patient 1 had changed from a happy to a frustrated child, crying often and expressing a lot of anger. She also developed a sleeping disorder waking up in the middle of the night and staying awake thereafter without any additional sleep during the day. Providing structure and sleep hygiene had no effect and different medication combinations, such as melatonin, hydroxyzine, pipamperone and pregabalin, had varying degrees of effect, but did not resolve the sleeping disorder. Her parents reported this as the most important burden of the disease for them.

No behavior or sleeping disorders were reported in patient 2.

### Assessment of treatment safety

#### Infusion associated reactions

Both patients exhibited infusion associated reactions on cerliponase alfa, patient 1 after the 15th infusion and patient 2 after the 6th infusion. In both patients these reactions were mild (Common Terminology Criteria for Adverse Events (CTCAE) grade 1 or 2) and included development of an exanthema/maculo-papular rash covering less than 30% of body surface area (CTCAE grade 2), fever up to 39.0 °C (CTCAE grade 1), nausea (CTCAE grade 1) and sinus tachycardia (CTCAE grade 1). In both patients these reactions could be well managed by adding corticosteroids (oral dexamethasone 0.15–0.4 mg/kg) to their premedication, with which these reactions did not reoccur. Similar reactions have been observed in patients after cerliponase alfa administration [15, 19]. No prolonged hospitalization was required. CSF cultures remained negative.

## Discussion

Classic late-infantile CLN2 disease is a rare rapidly progressive neurodegenerative disorder, resulting in early death. Enzyme replacement therapy with cerliponase alfa now makes it possible to treat patients with CLN2 disease. Schulz et al. [15] showed this therapy to be effective in slowing down disease progression. We evaluated treatment outcome after 2 years of treatment with cerliponase alfa in two siblings with CLN2 disease, both heterozygous for the two most common pathogenic *TPP1* variants and with the same environmental factors, and found cerliponase alfa not only to be effective in slowing down disease progression, but also in extending the presymptomatic phase of the disease.

Natural history studies have shown a homogeneous course of disease progression in CLN2 disease, reflected in a rapid decline in the combined motor and language score of the CLN2 CRS between age 3–6 years [4]. After start of ERT patient 1 had a slower disease progression than predicted based on natural history. She lost 1 point on the combined motor and language scale after 15 months of treatment with cerliponase alfa due to a regression in use of language. This is in line with the reported results in treated patients [15, 20]. The initial increase in CLN2 CRS total score was explained by a better control of her epilepsy, but fluctuated over time due to recurring grand mal seizures. This is why the CLN2 CRS combined motor and language score was chosen as the primary outcome measure over the CLN2 CRS total score in the pivotal trial [15], since cerliponase alfa is less likely to influence seizure control, as seizures result from existing neurological damage, or affect loss of vision as the retina will not be reached with intracerebroventricular administration. Her younger brother did not show any decline in score on the CLN2 CRS at age 4 years, where we would have expected him to become symptomatic around age 3–3.5 years [4], also based on symptom onset in his sister.

Although proven of great value in evaluating the clinical course in CLN2 disease and assessing treatment efficacy with cerliponase alfa [5], the CLN2 CRS is a rough scoring tool, in which a decline in score equals a large loss in functionality within that specific domain, and more subtle changes in functionality are not detected. In addition, the score could not capture an improvement in motor skills in patient 2 as he already exhibited the maximum score. We therefore chose to also evaluate the patients using domain specific tests. The GMFM-66 was used to evaluate motor function. This motor assessment tool was developed for children with cerebral palsy, but has been used to assess motor function in other disorders as well [21–24]. Although the symptoms in patients with CLN2 disease meet the criteria for the use of the GMFM-66 [25], no other studies have reported its use in this patient population. The motor impairment in patient 1 was clearly demonstrated in her GMFM-66 total score at baseline. Her score showed a small decline during 2 years of treatment, but was expected to show a more rapid decline based on the homogenous regression in motor function observed in untreated patients [4]. The GMFM-66 total score in patient 2 showed an age-appropriate increase (the maximum total score of 100 can only be reached from age 5 years onward without motor disabilities) during 2 years of treatment and was remarkably higher than that of his sister at the same age (Fig. 2). This clearly demonstrates efficacy of treatment with cerliponase alfa with respect to motor function.

The results from the neuropsychological evaluation were more difficult to interpret as these were influenced by motor function of patient 1, and lack of sufficient cooperation during testing of both patients. Therefore, it is plausible that they sometimes underperformed during testing given what was reported by their parents when the children were at home. Despite this a clear regression in developmental age was seen in patient 1, which aligned with both the loss in language score on the CLN2 CRS as well as the progression of cerebral and cerebellar atrophy on brain MRI. However, a more rapid cognitive decline was expected based on natural history data [4–6]. In patient 2 an increase in developmental age was seen. Although this was assessed not to be fully age-appropriate, and taking into account some degree of variability which can be seen in clinical phenotype between siblings with the same disorder, his neurocognitive development was much better than his sister at the same age where he was able to start regular primary school. His brain MRI showed mild periventricular signal changes, which can still be age-appropriate (terminal myelination zones) or, alternatively, may be a first sign of CLN2 as this has been described in infantile onset NCL [26], but not specifically in CLN2 patients. Follow-up brain MRI studies will be needed to make this discrimination. No cerebral or cerebellar atrophy was seen in patient 2 after 2 years of treatment, which is in sharp contrast with the brain MRI of his sister at a younger age (Fig. 3).

Cerliponase alfa is not expected to influence the onset of vision loss in CLN2 as it does not reach the retina with intracerebroventricular administration. No apparent visual problems were seen in both patients, although it should be noted that overt visual impairment occurs at a later stage in CLN2 disease [5]. Patient 1 had giant potentials and patient 2 had a prolonged P100 latency time on VEP studies. Both findings have been described in CLN2 patients [17], this could potentially be the first manifestation of CLN2 disease in patient 2.

The therapy was tolerated well by both patients. Although both had mild infusion associated reactions (CTCAE grade 2 at most), in accordance with reported adverse events [15, 16], these could be managed well with oral corticosteroids and never interfered with cerliponase alfa infusions.

## Conclusions

In conclusion, we show that intracerebroventricular ERT with cerliponase alfa can delay disease onset in CLN2 disease. In addition, the results in patient 1 confirm that cerliponase alfa slows down disease progression in CLN2 disease. Our and previously reported results [15, 20] demonstrate that intracerebroventricular ERT can have a significant impact on the disease course of CLN2 disease, as well as potentially other neurodegenerative lysosomal storage disorders, which is a breakthrough in the treatment of these severe disorders. Studies with a larger study population and longer follow up duration, like the ongoing phase 2 trial (NCT02678689), are needed to confirm our results and to establish long term treatment efficacy. If disease onset can be delayed for an extended period of time this may pave the road for possible inclusion of CLN2 disease in newborn screening programs and provide a bridge towards the development of innovative treatments, like gene therapy currently under development (NCT01161576; NCT00151216).

## Methods

### Treatment with cerliponase alfa

Both patients received a Rickham intracerebroventricular reservoir before they started treatment with biweekly intracerebroventricular infusions of 300 mg cerliponase alfa (Brineura®, Biomarin Pharmaceutical Inc.). We adhered to protocols for intracerebroventricular device access and infusion of cerliponase alfa [27, 28]. Antihistamines (clemastine 0.025 mg/kg) and paracetamol (20–30 mg/kg) were administered as premedication 30 min before each infusion. During administration, heart rate, oxygen saturation, blood pressure and temperature were monitored. During the first 7 infusions, both patients remained hospitalized overnight. Thereafter, infusions were given in a daycare setting where both patients were observed for an extra three hours after completion of the infusion.

### Assessment of treatment efficacy

#### CLN2 clinical rating scale

The CLN2 Clinical Rating Scale (CLN2 CRS) [5, 15] (“Appendix”) was used as the primary outcome measure in order to compare results with those reported in the first patient trial with cerliponase alfa [15]. We compared the sum score of the motor and language domains (max score 6; primary outcome measure of treatment efficacy in the clinical trial), as well as the total score of the four subdomains (max score 12) in both patients. The CLN2 CRS was administered by the same two clinicians (HHH, JMPH) every 3 months.

#### Evaluation of motor function

Next to the CLN2 Clinical Rating Scale motor function was evaluated by the same physiotherapist (LJG) every 3 months with the Gross Motor Function Measure-66 (GMFM-66) Dutch version [29]. The GMFM-66 consist of 66 items, divided into five categories (lying and rolling; sitting; crawling and kneeling; standing; walking, running, and jumping). Each item is scored on a four-point Likert scale. The instrument has been validated in children with cerebral palsy from 5 months to 16 years of age. In order to calculate a total score for the GMFM-66 a computer program, the Gross Motor Ability Estimator (GMAE), is needed with which individual item scores can be converted into an interval level total score. A 5-year old child without motor disabilities is able to reach the maximum score of 100. The GMFM-66 is based on the GMFM-88. The advantage of the GMFM-66 over the GMFM-88 is that children who are unwilling or unable to perform certain items can still be scored with an estimation of the scoring on that item based on the child’s pattern of responses to other items. This will then result in a GMFM-66 total score with an enlarged confidence interval [25, 30].

#### Neuropsychological outcome

Neuropsychological outcomes were evaluated at 6-month intervals by the same neuropsychologist (CK) with the Bayley Scales of Infant and Toddler Development, 3rd Edition, Dutch version (BSID-III-NL), as this is the age-appropriate test for children with a developmental age between age 1 and 42 months [31]. The patients were assessed with the cognitive and language scales, which are good predictors of preschool mental test performance. The motor scale was not used as motor function was assessed separately with the GMFM-66.

#### Neurological outcome

Epilepsy was evaluated continuously based on parental reports, and was assessed with the CLN2 CRS every 3 months. Anticonvulsant drugs were adjusted accordingly.

An ophthalmological examination was performed at baseline and after 2 years of treatment, to evaluate if there were signs of macular degeneration [32, 33]. Parents were asked about possible visual impairment every three months. As early markers for retinal damage, visual evoked potentials (VEP) and electroretinogram (ERG) were attempted every 6 months.

Both MRI brain studies of patient 1, as well as the follow-up MRI of patient 2, were performed on a 3 T MRI scanner. The imaging protocol included a 3D T1-weighted sequence, axial T2-weighted sequence and axial T2 Fluid Attenuated Inversion Recovery (FLAIR) sequence. In addition, Diffusion Weighted Imaging (DWI) and Susceptibility Weighted Imaging (SWI) was performed. No volumetric measurements were done. In patient 1, the baseline MRI was made at age 42 months and the follow-up MRI at age 73 months. The baseline MRI brain study of patient 2 at age 23 months was acquired on a 1.5 T MRI scanner using an imaging protocol for neuronavigation purposes only. His follow-up 3 T MRI scan was performed at age 48 months. All MRI studies were done under general anesthesia.

### Assessment of treatment safety

Both patients were monitored during and after treatment with cerliponase alfa (see ‘Treatment with cerliponase alfa’ section). Data regarding adverse events and any interventions needed to mitigate infusion associated reactions were recorded at every treatment. Adverse events were graded according to the Common Terminology Criteria for Adverse Events (CTCAE) toxicity scale, version 5.0 [34].

To monitor for possible bacterial colonization of the intracerebroventricular device and central nervous system infections, cerebrospinal fluid (CSF) was aspirated before every infusion and sent out for cell count, Gram staining and culturing up to 14 days after aspiration.

## Data Availability

Data sharing is not applicable to this article as no datasets were generated or analysed during the current study.

## Appendix

CLN2 clinical rating scale

**Table:**

Functional domain	Performance	Score
Motor function	Has grossly normal gait, no prominent ataxia, no pathologic falls	3
	Has independent gait as defined by ability to walk without support for 10 steps; obvious instability and possibly intermittent falls	2
	Requires external assistance to walk or can only crawl	1
	Can no longer walk or crawl	0
Language	Has apparently normal language that is intelligible and grossly age-appropriate, with no decline noted	3
	Has language that has recognizable abnormalities but includes some intelligible words; may form short sentences to convey concepts, requests, or needs	2
	Has language that is hard to understand with few intelligible words	1
	Has no intelligible words or vocalizations	0
Seizures (grand mal)	No seizures per 3-month period	3
	1 to 2 seizures per 3-month period	2
	1 seizure per month	1
	> 1 seizure per month	0
Visual function	Recognizes desirable object, grabs at it	3
	Grabbing for objects uncoordinated	2
	Reacts to light	1
	No reaction to visual stimuli	0

## Abbreviations

TPP1: Tripeptidyl peptidase
CLN2 CRS: CLN2 clinical rating scale
GMFM-66: Gross motor function measure-66
BSID-III-NL: Bayley Scales of Infant and Toddler Development, 3rd Edition, Dutch
VEP: Visual evoked potentials
ERG: Electroretinogram
MRI: Magnetic resonance imaging
CTCAE: Common terminology criteria for adverse events
CSF: Cerebrospinal fluid
FLAIR: Fluid attenuated inversion recovery
DWI: Diffusion weighted imaging
SWI: Susceptibility weighted imaging
